# 3D Evaluation of material-dependent volumetric and interfacial changes in CAD/CAM partial crowns: a semi-automated micro-CT approach

**DOI:** 10.3389/fdmed.2026.1843676

**Published:** 2026-08-18

**Authors:** Ellen Schulz-Kornas, Olivia Giesa, Tobias Meißner, Nadia Oberück, Philipp D. Lösel, Stephan Handschuh, Dirk Ziebolz, Rainer Haak

**Affiliations:** 1 Department of Cariology, Endodontology and Periodontology, Leipzig University, Leipzig, Germany; 2 Department of Materials Physics, The Australian National University, Canberra, ACT, Australia; 3 VetCore Facility for Research/Imaging Unit, University of Veterinary Medicine Vienna, Vienna, Austria; 4 Department of Conservative Dentistry and Periodontology, Brandenburg Medical School Theodor Fontane (MHB), Brandenburg an der Havel, Germany; 5 Institute of General Medicine, Faculty of Medicine, Leipzig University, Leipzig, Germany

**Keywords:** CAD/CAM, ceramics, contact surface, micro-CT, partial crown, resin-based composite, segmentation, volume

## Abstract

**Objectives:**

Changes in the 3D volumes, interfaces, and interfacial gap formation were examined of adhesively luted lithium disilicate (LS2) and resin-based composite (RBC) partial crowns during artificial aging and imaged by micro-computed tomography (micro-CT).

**Methods:**

Sixteen human mandibular molars were restored using either 8 CAD/CAM partial crowns made of LS2 (IPS e.max® CAD) or RBC (Tetric® CAD). After adhesive luting (Variolink® Esthetic DC, adhesive luting composite), artificial aging [water storage for 90 days (t1) and thermo-mechanical loading (t2)], the partial crowns were analyzed by micro-CT (Avizo, Biomedisa). Paired *t*-test, ANOVA and Tukey HSD test (*α* = 0.05) were used to analyze area and volume parameters; e.g., luting space volume (VLS), luting space thickness (TLS), and partial crown contact area to the cavity and tooth tissues (CPT).

**Results:**

Across artificial aging, most area parameters and all four volume parameters remained stable in both materials. Yet, there were changing values in areas of direct contact between the partial crown material and the tooth tissues: CPE, CPT were generally smaller at t1 for LS2 (2.2 ± 1.2%) compared to RBC (4.5 ± 1.3%), especially at the inflection and saddle points of the round preparation form. Across both time points, LS2 showed consistently higher mean luting space thickness than RBC. It remained without gap progression.

**Conclusions:**

The semi-automated 3D micro-CT analysis enables a comprehensive evaluation of adhesive interfaces and may help to develop new ideas to enhance long-term restoration outcomes. RBC partial crowns had more surfaces in direct contact with the cavity floor than LS2 partial crowns.

**Clinical significance:**

The approach with Biomedisa has potential translational value and may enable individualized quality control and long-term monitoring of adhesive restorations in clinical practice. Both materials achieve comparable survival rates, wear resistance, and interface stability under simulated conditions and when minimum material thickness is respected.

## 1 Introduction

Partial crowns (PCs) fabricated via CAD/CAM technology have become a clinically established treatment option for restoring structurally compromised posterior teeth since the mid-1980s (1, 2). One of the key advantages of CAD/CAM is its ability to quickly and precisely replicate the natural anatomy of teeth, ensuring an optimal fit and function for the patient. In contrast to full crowns, adhesively luted PCs enable a minimally invasive approach while maintaining both functional and aesthetic outcomes (3). They have been described as superior to crowns in resisting fatigue stress with decreasing material thickness (4). Advances in adhesive luting have led to preparation designs with rounded, non-retentive geometries that preserve more hard tissue while relying on the adhesive interface for stability (5).

Lithium disilicate (LS2) and resin-based composites (RBC) are two clinically established materials for CAD/CAM PC fabrication, offering distinct mechanical properties (6, 7). LS2 has a high modulus of elasticity (95 GPa) similar to that of enamel (86 GPa) in contrast to RBC (10 GPa) which is closer to dentin (15 GPa). Unlike LS2, RBCs show viscoelastic behavior, allowing for plastic deformation under functional forces (8, 9) and display comparable fracture strengths to LS2 in molar restorations (10). However, there are only a few *in vitro* studies comparing both materials when luted to extracted teeth before and after thermomechanical loading (TCML) (11), especially for PCs.

While there are defined terms and measurements for the marginal gap and a clinically recommended upper limit of 120 µm (12) there is no uniform terminology regarding the luting space [i.e., internal/vertical/occlusal gap (13), discrepancies (14), adaptation (15), cement thickness (16), misfit (17), space (18)] which leads to confusion and makes them hard to compare. Various scientific approaches have been reported for analyzing luting space thickness in two and three dimensions, although a clinical suggestion for PC is still lacking (19). In reports where both marginal gap and mean luting space thickness were compared, the value of the latter was always larger (19).

In addition to the definition by Krämer et al. (20) the luting space consists not only of the adhesive luting composite (ALC) and the adhesive but also of voids. Examples for voids are gaps at the interfaces between two different materials and pores**.** Thickness and distribution of ALC within the luting space influence the mechanical integrity and long-term performance of the restoration (20). ALC can compensate for stress peaks due to its material properties (21, 22). Thin and uniform layers of ALC are considered beneficial (23). However, studies have found that luting space thickness in PCs varies greatly, typically ranging from 23 to over 400 µm (24). Thicker luting space is often found in the central grove, at the tip of the preparation, or along the axial walls (25). It remains unclear which ALC material provides the thinnest and most uniform luting space, as the outcome depends on factors such as the preparation design, the scanner (14) and milling unit, bur type, chosen luting space settings (19), and whether the measurement is taken before or after cementation or adhesive luting of the restorations.

Thick luting space results in a greater volume change of ALC after light curing, leading possibly to greater stress and leakage (26). Especially for rounded, non-retentive PC preparations, there is a lack of data on the spatial distribution and behavior of ALC within the luting space and the interfaces to tooth and restorative materials. Initial evidence suggests that, particularly at the composite interface and around the margins of the PC, gap formation is the first step toward failure (27). Quantitative 3D imaging could provide information about where a failure originates and how it spreads.

Micro-computed tomography (micro-CT) enables high-resolution, non-destructive 3D visualization of internal interfaces, providing valuable insights into luting space morphology and interfacial adaptation before and after aging with high accuracy (28–30). In order to evaluate the data from the scans, they must first be segmented into their different materials. However, the segmentation of micro-CT datasets of biological specimens is time-consuming, requiring manual labeling steps that limit the scalability of the study (31). In dental research, thresholding or automatic segmentation approaches often fail to differentiate adjacent materials accurately (32, 33). To improve the efficacy of manual image segmentation, a semi-automatic segmentation application, Biomedisa, was developed (31). Biomedisa stands out because it is a validated open-source, easy-to-use repeatable interpolation algorithm that preserves manual segmentation accuracy (31), allowing for the use of centralized IT facilities and significantly reducing the number of sections that need to be segmented manually. The present study aimed to investigate material-dependent differences in volume, area, and interfacial morphology of LS2 and RBC partial crowns, as well as their respective luting space thickness and volume. It will be analysed before and after TCML, to quantify material-dependent structural parameters of the PCs in 3D.

It was hypothesized that:

(a) Material volume/interface area changes and voids would increase after artificial aging,
(b) with smaller material-dependent changes expected in LS2 than in RBC (LS2 < RBC) due to its material properties (e.g.: E-modulus).

## 2 Material and methods

### 2.1 Specimen preparation

**Figure 1:**
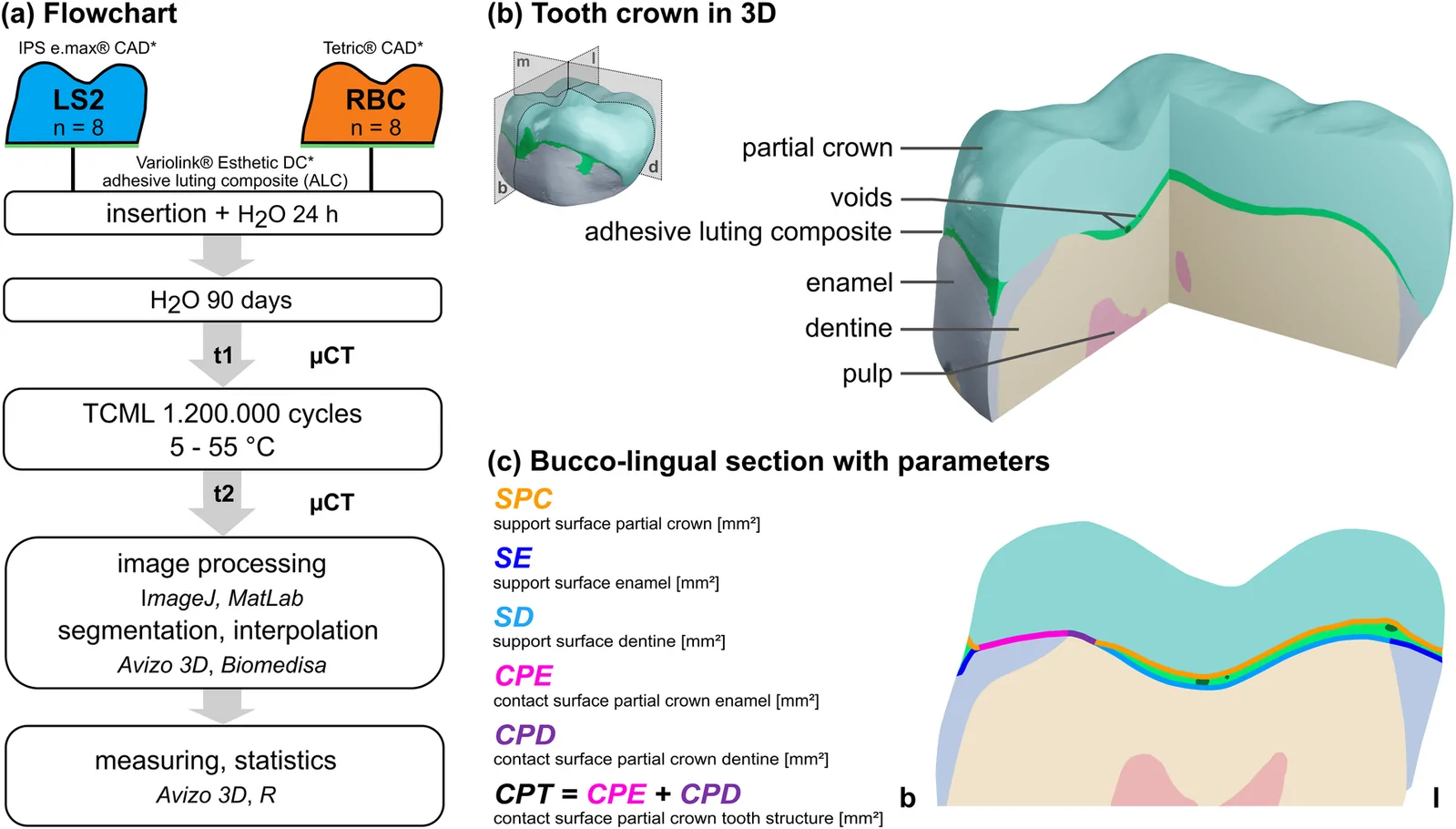
**(a)** Flowchart of the experimental design for analyzing material performance via micro-CT LS2., lithium disilicate ceramic; RBC, resin-based composite; AA, artificial aging: H_2_O, water storage; TCML, thermocycling-mechanical loading); *product names; **(b)** schematic 3D illustrations of an adhesively luted partial crown, enlarged and sectioned to show the internal structure, including restoration and tooth materials; **(c)** bucco (b)-lingual (l) section and the measured surface parameters.

Sixteen extracted caries-free human lower molars were collected with patient consent and stored in a 0.5% chloramine-T solution at 4 °C to be used within six months. The root growth was completed, and the root canal system was untreated. The teeth were randomly assigned to two groups (*n* = 8) LS2 or RBC (Figure 1, materials listed in Table 1). The same sixteen lower molars were used in Oberück et al. (27); in Oberück et al. (27) the method applied was OCT and in the current study it was micro-CT only.

**Table 1:** Composition, mechanical properties, and pre-treatment of the investigated materials according to manufacturer's recommendation.

Code	Classification	Minimal material thickness [mm]	Product name	Composition [wt%]		Water sorption [µg/mm^3^]/7 days	Density [g/cm^3^]	E-modulus [GPa]
LS2	Lithium disilicate ceramic	1.5	IPS e.max® CAD^a^	SiO_2_	57–80	0	2.50 ± 0.10	95
				Li_2_O	11–19			
				K_2_O	0–3			
				P_2_O_5_	0–1			
				ZrO_2_	0–8			
				ZnO	0–8			
				Al_2_O_3_	0–5			
				MgO	0–5			
				coloring oxides	0–8			
RBC	Resin-based composite	1.0	Tetric® CAD^a^	nanohybrid filler	86	22.5	1.96	10
				UDMA, TEGDMA	14			
ALC	Adhesive luting composite	≤ 0.05	Variolink® Esthetic DC^a^	UDMA + BDDMA	≤ 20	≤ 40	1.86	0.1
				inorganic filler + YbF_3_	38			

UDMA, urethane dimethacrylate, TEGDMA, triethylene glycol dimethacrylate, BDDMA, butandiol dimethacrylate.

a Ivoclar Vivadent (Schaan, Liechtenstein).

### 2.2 Restorative procedure

As part of quality assurance, each preparation was evaluated by an experienced, calibrated dentist, and minor adjustments were made when necessary to ensure consistency across all specimens. The teeth were prepared using magnification loupes (magnification: 2.8x) and diamond burs adjusted for the preparation of partial crowns (expert-set ceramic inlays and partial crowns 4,562, Komet Dental/GEBR. BRASSELER GmbH & Co. KG, Lemgo, Germany). Occlusal reduction of the cusps was 2 mm with a buccal and lingual preparation margin set above the anatomic tooth equator following the guideline for indirect ceramic restorations (5). Approximal reduction was 4 mm, ensuring the preparation border was positioned above the enamel-cement junction. A second calibrated dentist examined the prepared teeth to assess the quality of the preparation.

Specimens were digitized with an intraoral scanner (CEREC Primescan, Cerec 5, Dentsply Sirona Inc., Pennsylvania, USA). The PCs were manually designed (inLab CAM, version 20.0.1.203841, Sirona Dental Systems GmbH, Bensheim, Germany), reducing the anatomic structure to standardize a maximum occlusal layer thickness of 3.5 mm in cusp areas. The luting space was set to 80 µm, and minimal occlusal layer thickness was milled at 1.5 mm (CEREC MC XL, Sirona Dental Systems GmbH, Bensheim, Germany) using block size C14 (A3/HT). For LS2, PCs were degreased in 70% ethanol, and the internal surface was etched with 4% hydrofluoric acid (IPS Ceramic Etching Gel, Ivoclar Vivadent, Schaan, Liechtenstein) for 20 s. A universal silane (Monobond Plus Universal Primer, Ivoclar Vivadent, Schaan, Liechtenstein) was applied for 60 s and air-dried. For RBC, the inner surface of the PCs was blasted with 50 µm aluminum oxide at a pressure of 1.5 bar, cleaned in 70% ethanol in an ultrasonic bath, air-dried, and conditioned for 20 s with the universal adhesive (Adhese Universal, Ivoclar Vivadent, Schaan, Liechtenstein). Prepared tooth surfaces were conditioned with phosphoric acid (30 s for enamel, 15 s for dentin, Total Etch 37% phosphoric acid, Ivoclar Vivadent, Schaan, Liechtenstein), then conditioned with the universal adhesive (Adhese Universal VivaPen, Ivoclar Vivadent, Schaan, Liechtenstein) and light-cured for 20 s. The ALC (Variolink Esthetic DC, Ivoclar Vivadent, Schaan, Liechtenstein) was applied to all PCs. The restorations were then positioned with finger pressure, initially light-cured for 2 s from each side, and superfluous ALC was removed using a scaler. Light curing was completed after applying a glycerine gel (Liquid Strip, Ivoclar Vivadent, Schaan, Liechtenstein) for 20 s from each side. Restoration margins were polished to high gloss (Optra Gloss, Ivoclar Vivadent, Schaan, Liechtenstein).

### 2.3 Artificial aging

After restoration, specimens were stored in deionized water at 37  °C for 90 days for water absorption to reach its equilibrium (9, 34) (WTB Binder MB6, Tuttlingen, Germany). For artificial aging, thermo-mechanical loading (TCML) was performed in a chewing simulator (CS 4.8, SD Mechatronics, Feldkirchen-Westerham, Germany). To simulate the periodontal ligament, specimens were coated with a 1 mm layer of polyether (Impregum™, 3 M Espe, Seefeld, Germany), using a wax spacer (Wapfeno, VEB Wachswarenfabrik, Nordhausen, Germany) for uniform thickness, and embedded in cold-polymerizing resin (Technovit® 4,000, Kulzer GmbH, Wehrheim, Germany). A 3D-printed fixture ensured standardized load transfer. TCML was executed using 5 × 600 thermal cycles (5  °C/55  °C) combined with 1.2 × 10^6^ mechanical loading cycles at 50 N and 6 mm/s (1.6 Hz), generating 0.5 mm horizontal excursion. At the end of each thermal-cycling or mechanical loading sequence, the specimens were continuously rinsed with water at 37  °C for the remainder of the aging process to prevent drying. Steatite antagonists (6 mm diameter; CeramTec, Plochingen, Germany) were used.

The SD-OCT used in our previous study (27) on the same samples has a near-infrared light (Telesto-II Sp21, wavelength: 1,550 nm, 28 kHz, sensitivity ≤106 dB; power on sample 4.5 mW; spot size 22.5 µm, version 5.4.2.0; Thorlabs, Dachau, Germany), which was originally used in ophthalmology, indicating that it is unlikely to damage even a very fragile human retina. Therefore, SD-OCT is considered a very gentle approach due to its non-ionization character. It has an extremely promising diagnostic power; e.g., as an alternative to conventional imaging techniques like radiography [for comparison see Rigotti et al. (35), Strumpski et al. (36), Braga et al. 37]. Furthermore, the SD-OCT measurements in Oberück et al. (27) were completed in just a few seconds and under moist conditions, keeping the samples in a stable condition, thus, no effects on the artificial aging procedure were expected.

### 2.4 Micro-computed tomography (micro-CT)

The specimens were imaged at 91 days after adhesive luting and water storage (baseline = t1) and after TCML with 1,200,000 cycles (t2). High-resolution micro-CT (SkyScan 1,172, Bruker, micro-CT, Kontich, Belgium) scans were made with the following settings: 100 kV, 100 µA, 2,600 ms of exposure, aluminum/copper filter (500/40 µm), 180° rotation, angular increments between projections 0.2°, image pixel size 3.47–4.09 µm (depending on tooth size), frame averaging 4, random movement 10 with an average scanning time of 260 min per scan. 3D reconstructions of the micro-CT images were done using NRecon 1.7.4.6 (Bruker, micro-CT, Kontich, Belgien, maximum ring correction level 50, Hounsfield unit minimum −1,000 maximum +6,000) to generate 16-bit TIFF image stacks.

### 2.5 Micro-CT image processing and segmentation

Segmentation was done by one trained operator on the same workstation and system. Raw micro-CT data were converted from 16-bit to 8-bit using ImageJ (v1.53c, NIH, Bethesda, MD, USA). Peripheral air voxels were cropped out via a Matlab script (R2022b, The MathWorks Inc., Natick, MA, USA) to minimize file size. The digital workflow is given in detail in Supplementary S1 (digital workflow and technical specifications in Supplementary S1a,b; and the step-wise procedure for the semi-automated segmentation and its specifications in Supplementary S1c). Examples of unfiltered micro-CT images are given in Supplementary S2. Segmentation was performed in Avizo 3D (v2021.1, Thermo Fisher Scientific, Waltham, MA, USA) by defining a region of interest (ROI) extending 20 xy-slices beyond the visible edges of restorative material. Each dataset contained approximately 2,000 xy-image slices per ROI. Lösel et al. (31) showed exemplarily that for manual segmentation of biological samples approximately 20% of all slices need to be labeled, while for the Biomedisa approach it was only about 3%. In the current study a compromise between accuracy and workload was chosen to label about 5% equidistant slices. Every 20th slice was manually labeled to differentiate six materials (PC, luting composite, enamel, dentine, pulp, exterior). This took about 17 h per tooth and time point. Artifacts (<100 pixels) were excluded before full-volume interpolation using Biomedisa (31). This application first utilizes manual segmentation to provide information on the dataset, and then uses a random walk algorithm to interpolate the data, considering the complete underlying image data (38, 39). After cleaning the fully labeled dataset using a threshold approach, voids were segmented in the luting space ROI.

### 2.6 Material statistics

Volume and surface data were analyzed with Avizo 3D (Thermo Fisher Scientific, Waltham, MA, USA). Surface reconstruction was performed using the “Generate Surface” module (compactify enabled, minimum edge length set to 1, repeatable algorithm, and smoothing extent set to 5). Surfaces and interfacial regions were extracted using the “Surface Area Volume” module, and volumes were determined using “Material Statistics”. The thickness of the luting space (TLS) was assessed at every possible 3D measurement point within the luting space using “Thickness map” and “Histogram” modules. Twelve quantitative parameters characterizing hard tissue and restoration surfaces (SPC, SE, SD, ST), restorative interfaces (CPE, CPD, CPTR), luting space thickness (TLS), and volumes (VLS, VV, VPC, VA) were analyzed (refer to Table 2).

**Table 2:** Parameter use, acronyms, descriptions, units, feature, and related aims and hypotheses; selected parameters for inferential statistical analyses are set in bold.

**Acronym**	**Parameter**	**Description**	**Unit**	**Feature**	**Aims and hypotheses**
SPC	Support surface partial crown	Surface area of inner crown support surface; necessary for calculation of CPE, CPD, CPT	[mm^2^]	Area	3D description
SE	Support surface enamel	Surface area of prepared enamel surface; necessary for calculation of ST	[mm^2^]		3D description
SD	Support surface dentine	Surface area of prepared dentin surface; necessary for calculation of ST	[mm^2^]		3D description
ST	Support surface tooth	Surface area of prepared tooth; SE + SD = ST; necessary for calculation of CPE, CPD, CPT, CPTR	[mm^2^]		3D description
**CPE**	Contact surface partial crown enamel	Surface area of direct contact surface between crown and enamel	[mm^2^]		3D description, area
**CPD**	Contact surface partial crown dentine	Surface area of direct contact surface between crown and dentine	[mm^2^]		3D description, area
**CPTR**	Contact surface partial crown tooth	Contact surface between crown and tooth relative to prepared tooth; CPT = CPE + CPD CPTR = (CPT/ST) * 100	[%]		3D description, area
**TLS**	Thickness luting space	Mean of luting space thickness incl. luting composite and voids	[µm]	Thickness	3D description, thickness
**VLS**	Volume luting space	Volume of luting space incl. volume luting composite and voids	[mm^3^]	Volume	3D description, volume and area
VV	Volume voids	Volume of the voids	[mm^3^]		3D description
VPC	Volume partial crown	Volume of the partial crown materials	[mm^3^]		3D description, volume and area
**VA**	Volume abrasion	Volume of the material loss	[mm^3^]		3D description, volume and area

### 2.7 Statistical analysis

The open-source software R (version 4.5.2) was used for data processing and statistical analyses. The R packages used are listed in the Supplementary S3 and R code in Supplementary S4. Surface, thickness, and volume parameters were explored by calculating means, medians, interquartile ranges and visualized with strip charts. Normality was assessed by residual plots and the Shapiro–Wilk test. Levene's test was applied to evaluate the homogeneity of variances. As all continuous variables were normally distributed, independent *t*-test was used for group comparisons and paired *t*-test was used for longitudinal analysis. Cohen's d effect size was calculated for each contrast. The level of significance was set to *α* = 0.05. The present exploratory micro-CT study is based on the sample set of a former OCT study (27), which showed adequate power and sample size in detecting clinically relevant differences in restoration interfaces using optical coherence tomography. Thus, no additional *a priori* power analysis was conducted. Instead, the statistical sensitivity of this study was characterized with an independent imaging modality, and *post-hoc* power analyses were performed for six selected micro-CT parameters (CPE, CPD, CPTR, TLS, VLS, VPC, Table 2). For independent *t*-tests with *n* = 8 per group (*α* = 0.05, power = 0.80), the minimum detectable Cohen's effect size was d = 1.44, corresponding to very large effects. For paired *t*-tests with *n* = 8 pairs, the minimum detectable effect was d = 1.20. Achieved power was calculated for each comparison based on observed effect sizes and exceeded a power for CPE and CPTR of >90%.

## 3 Results

### 3.1 Segmentation quality and image characteristics

The interpolation accuracy and edge detection were visibly high compared to the manually labeled slices at both time points, with exception of low-contrast interfaces, where even visual differentiation was difficult. Compared to the RBC group, the LS2 micro-CT images showed larger image contrast between the restorative materials. Voids such as pores within the luting space and gaps at the interfaces could not be detected sufficiently by Biomedisa. These were segmented later using a threshold approach.

### 3.2 Survival and 3D observations

**Figure 2:**
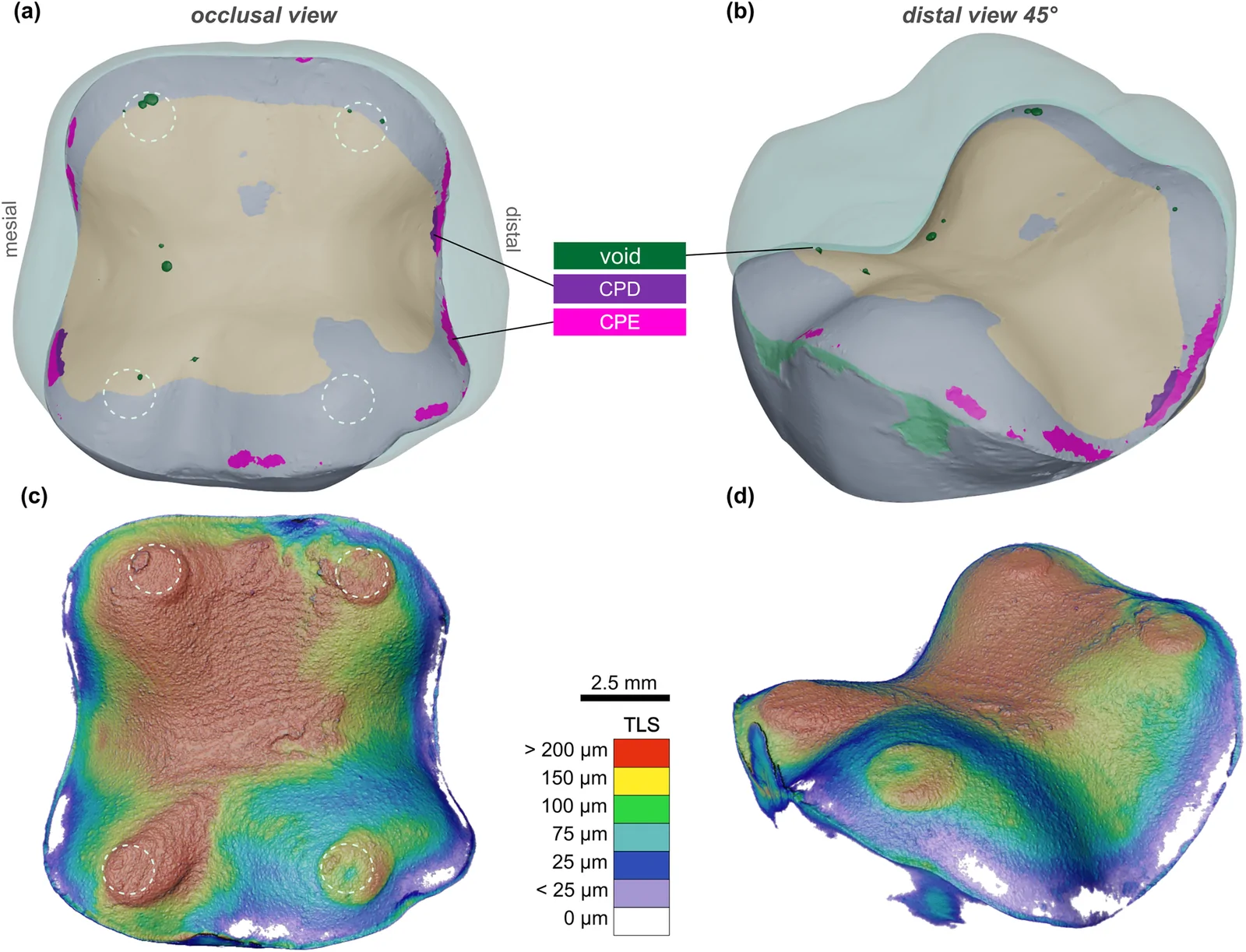
3D model of the partial crown (t1), the prepared surface (lower molar) from occlusal **(a)** and 45° distal view **(b)** showing the location of voids and direct contact surfaces to the partial crown within dentine (CPD, contact surface partial crown dentine) and enamel (CPE, contact surface partial crown enamel) support surface. Distribution of the luting composite (c and d) indicating different material thicknesses (TLS); drill compensation in **(a)** and **(c)** given as a grey dotted line.

All restorations survived water storage and TCML without visible fractures, chippings, or cracks (hypothesis a). In both groups, the luting space including voids and contact areas between the partial crown and enamel (CPE) or dentine (CPD) was clearly visible and could be distinguished by micro-CT (Figure 2, Supplementary S3). Areas of direct contact between the partial crown material and the adjacent tooth structure appeared in particular at the approximal inflection and saddle points of the rounded surface of the preparation. Enamel makes up most of CPT (LS2 t1: 90.24 % t2: 89.22 %, RBC t1: 97.41 % t2: 97.78 %). Voids appeared in drill compensation areas and centrally in the valleys between the cusps (Figure 2). No gap progression at the interfaces was observed in either group. Almost all specimens (in total: 14 out of 16, 7 per group) showed residual enamel islands in the center of the support surface (Figures 2a,b). ALC makes up 99.7% of the luting space.

### 3.3 Area, volume, and thickness changes during aging

**Figure 3:**
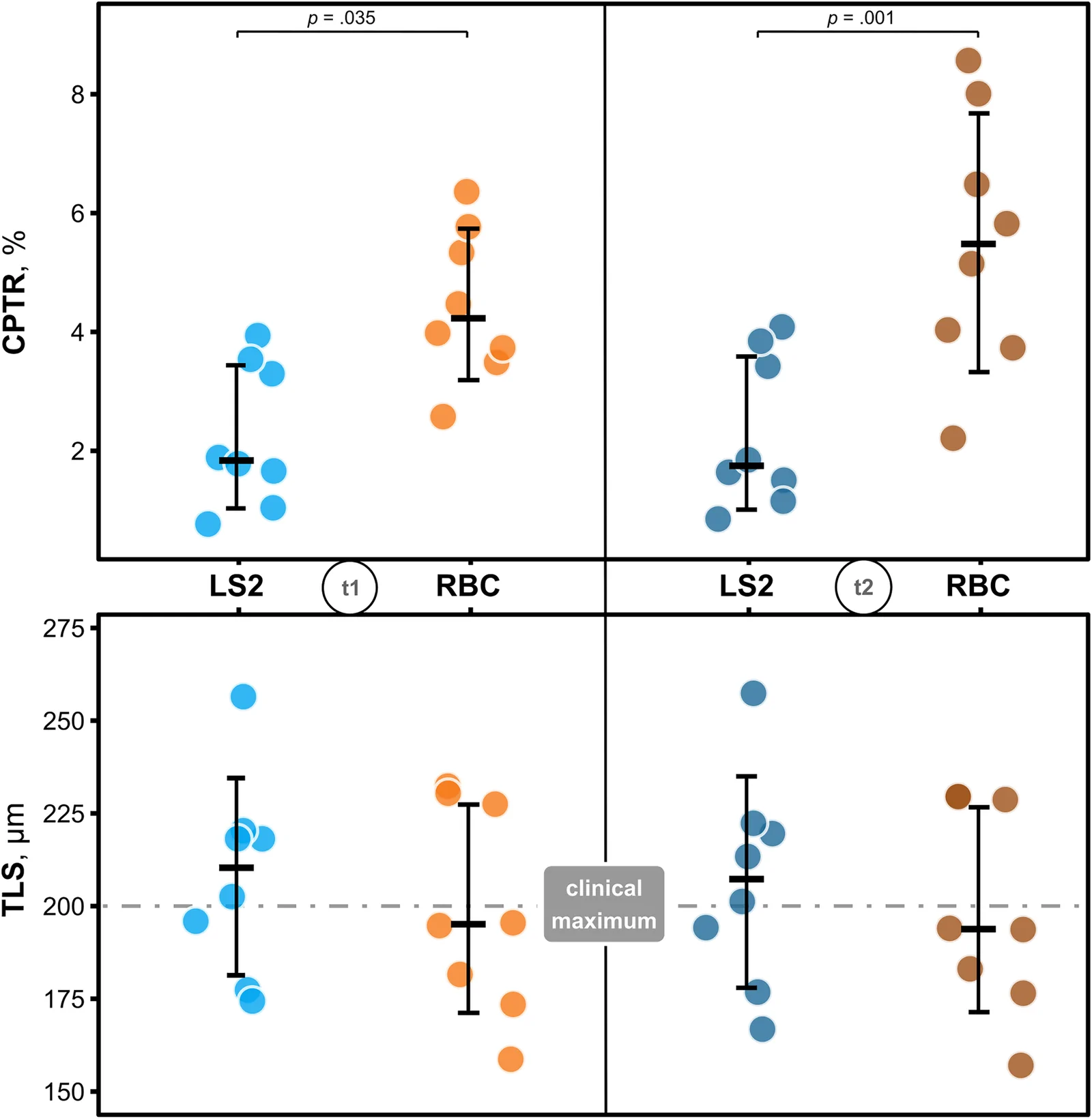
Scatterplots of the relative contact surface of the partial crown to the tooth (CPTR) and of the thickness of the ALC (TLS) at the two points in time t1 and t2 as well as the two materials (pooled data for all teeth per group) LS2: IPS e.max CAD, RBC: Tetric CAD. The clinical maximum of TLS was set to 200 µm as a compromise for a desired target value for the thickness of the luting space.

Across artificial aging, four of six selected parameters remained stable (Table 3: CPE, CPD, CPTR, TLS). Although VLS and VPC reached statistical significance (*p* = 0.03 and *p* = 0.004, respectively), effect sizes were negligible (d = 0.04 and d = 0.06), indicating no clinically meaningful change. Significant differences between LS2 and RBC were consistently observed at both timepoints at the contact areas between the partial crown material and the adjacent tooth structure (Table 3): CPE and CPT showed the largest effects at t1 (d = −2.41 and −2.16, respectively) and remained similarly pronounced at t2 (d = −2.1 and −2.01), with all comparisons reaching statistical significance (*p* ≤ 0.002). CPTR followed the same pattern with large effect sizes at both timepoints (d = 1.8, *p* ≤ 0.004), while CPD showed no meaningful difference between materials (d = −1.5, *p* > 0.82). LS2 partial crowns had a significantly smaller relative and absolute partial crown to tooth contact area (CPT, CPTR) at both time points (t1 and t2, Figure 3, Table 3).

**Table 3:** Descriptive (mean and standard deviation) and comparative statistics (independent *t*-test, paired *t*-test, as given for the parameters.

Parameter	LS2 t1 vs. *LS2 t2*			LS2 t1 vs. *RBC t1*			RBC t1 vs. *RBC t2*			LS2 t2 vs. *RBC t2*		
	mean + SD	*p*-value	Cohens d	mean + SD	*p*-value	Cohens d	mean + SD	*p*-value	Cohens d	mean + SD	*p*-value	Cohens d
SPC [mm^2^]	134.50 ± 21.00 vs. *37.39* *±* *23.45*	0.15	−0.11	134.50 ± 21.00 vs. *133.40* *±* *13.93*	0.9	0.06	133.40 ± 13.93 vs. *136.16* *±* *14.18*	0.003	−0.19	137.39 ± 23.45 vs. *136.16* *±* *14.18*	0.9	0.06
SE [mm^2^]	62.55 ± 13.56 vs. *63.77* *±* *15.90*	0.26	−0.05	62.55 ± 13.56 vs. 72.27 ± 14.08	0.18	−0.7	72.27 ± 14.08 vs. *71.44* *±* *16.02*	0.47	0.04	63.77 ± 15.90 vs. *71.44* *±* *16.02*	0.35	−0.48
SD [mm^2^]	64.1 ± 16.47 vs. *64.57* *±* *16.43*	0.15	−0.03	64.1 ± 16.47 vs. *53.5* *±* *21.35*	0.29	0.56	53.5 ± 21.35 vs. *53.42* *±* *21.13*	0.5	0.003	64.57 ± 16.43 vs. *53.42* *±* *21.13*	0.26	0.59
ST [mm^2^]	126.64 ± 19.55 vs. *128.33* *±* *21.17*	0.21	−0.07	126.64 ± 19.55 vs. *125.77* *±* *17.59*	0.93	0.05	125.77 ± 17.59 vs. *124.86* *±* *15.73*	0.47	0.05	128.33 ± 21.17 vs. *124.86* *±* *15.73*	0.72	0.19
**CPE** [mm^2^]	2.45 ± 0.99 vs. *2.52* *±* *1.24*	0.68	−0.05	2.45 ± 0.99 vs. *5.36* *±* *1.39*	0.0004	−2.41	5.36 ± 1.39 vs. *6.57* *±* *2.40*	0.11	−0.55	2.52 ± 1.24 vs. *6.57* *±* *2.40*	0.002	−2.1
**CPD** [mm^2^]	0.28 ± 0.36 vs. *0.33* *±* *0.36*	0.3	−0.13	0.28 ± 0.36 vs. *0.34* *±* *0.36*	0.82	−0.15	0.34 ± 0.36 vs. *0.33* *±* *0.30*	0.93	0.08	0.33 ± 0.36 vs. *0.33* *±* *0.30*	0.99	−0.004
CPT [mm^2^]	2.70 ± 1.18 vs. *2.81* *±* *1.36*	0.55	−0.08	2.70 ± 1.18 vs. *5.53* *±* *1.42*	0.0007	−2.16	5.53 ± 1.42 vs. *6.73* *±* *2.42*	0.13	−0.56	2.81 ± 1.36 vs. *6.73* *±* *2.42*	0.002	−2.01
**CPTR** [%]	2.24 ± 1.20 vs. *2.30* *±* *1.29*	0.7	−0.04	2.24 ± 1.20 vs. *4.46* *±* *1.27*	0.003	−1.8	4.46 ± 1.27 vs. *5.50* *±* *2.18*	0.12	−0.52	2.30 ± 1.29 vs. *5.50* *±* *2.1*	0.004	−1.79
**TLS** [µm]	213.44 ± 80.35 vs. *212.90* *±* *81.33*	0.24	0.04	213.44 ± 80.35 vs. *204.42* *±* *83.21*	0.54	0.32	204.42 ± 83.21 vs. *205.67* *±* *83.99*	0.72	0.01	212.90 ± 81.33 vs. *205.67* *±* *83.99*	0.6	0.27
**VLS** [mm^3^]	21.11 ± 5.33 vs. *20.91* *±* *5.35*	0.03	0.04	21.11 ± 5.33 vs. *19.23* *±* *3.94*	0.44	0.4	19.23 ± 3.94. vs. *19.02* *±* *3.88*	0.05	0.05	20.91 ± 5.35 vs. *19.02* *±* *3.88*	0.44	0.4
VV [mm^3^]	0.06 ± 0.03 vs. *0.06* *±* *0.04*	0.22	0.06	0.06 ± 0.03 vs. *0.07* *±* *0.07*	0.79	−0.14	0.07 ± 0.07 vs. *0.06* *±* *0.07*	0.23	0.03	0.06 ± 0.04 vs. *0.06* *±* *0.07*	0.79	−0.14
VPC [mm^3^]	233.55 ± 40.79 vs. *230.86* *±* *40.19*	0.004	0.06	233.55 ± 40.79 vs. *212.53* *±* *25.99*	0.24	0.61	212.53 ± 25.99 vs. *213.71* *±* *26.14*	0.09	−0.05	230.86 ± 40.19 vs. *213.71* *±* *26.14*	0.33	0.51
**VA** [mm^3^]	NA	NA	NA	NA	NA	NA	NA	NA	NA	2.06 ± 0.79 vs. *1.61* *±* *0.30*	0.16	0.76

SPC, support surface partial crown; SE, support surface enamel; ST, support surface tooth; CPE, contact surface partial crown enamel; CPD, contact surface partial crown dentine; CPTR, contact surface partial crown tooth; TLS, thickness luting space; VLS, volume luting space; VV, volume voids; VPC, volume partial crown; VA, volume abrasion; materials LS2, lithium disilicate ceramic; RBC, resin-based composite; time points (t1, t2), selected parameters for inferential statistical analyses are set in bold.

Across both time points, LS2 and RBC showed similar thickness patterns: at t1, LS2 exhibited a mean thickness of 207.9 µm (SD = 26.6 µm), while RBC reached 199.3 µm (SD = 28.1 µm). This pattern remained stable at t2, with mean values of 206.5 µm (SD = 28.5 µm) for LS2 and 199.0 µm (SD = 27.6 µm) for RBC. Across all pairwise contrasts between materials, no statistically significant differences in mean luting space thickness were detected (all *p*  > 0.54). Cohen's d values ranged from 0.27 to 0.32, corresponding to negligible to small effects. Most areas above 200 µm are located centrally in between the cusps of the preparation and in the areas of the drill compensation (Figures 2c,d). Thus, there is an absence of a detectable difference in both materials, with comparable thickness distributions, and no clinically relevant differences could be derived (Figure 3). No significant plastic deformation or volume loss (VA, *p* = 0.163, d = 0.76, Table 3) was observed in either material.

## 4 Discussion

This study investigated material-dependent volumetric and interfacial changes in lithium disilicate (LS2) and resin-based composite (RBC) partial crowns (PC) subjected to artificial aging using Biomedisa for segmentation. Despite artificial aging, both materials demonstrated high volumetric similarity, minimal abrasive wear and stable adhesive interfaces.

Although micro-CT generates volumetric data, most studies rely on 2D linear measurements derived from virtual sections without any segmentation (19, 40). Only a few studies have fully utilized the potential of 3D measurements, providing results from 3D space (mm^3^) and 2D linear (µm) measurements (41, 42). Traditional micro-CT segmentation approaches rely either on manual segmentation and linear interpolation or global thresholding, both of which have considerable limitations (32). Manual segmentation can be highly accurate and labor-intensive when following a standard protocol performed by an expert in the field and is thus not scalable to large datasets. Threshold-based automation struggles with low-contrast interfaces between adjacent materials. By combining sparse manual pre-segmentation with algorithm-based interpolation, Biomedisa provided data in a manageable amount of time. For comprehensive quantification of volumetric (VPC, VLS, VV), surface (ST, SPC, CPTR), and thickness (TLS) parameters across large datasets, these metrics were rarely fully quantified in prior micro-CT studies on adhesive restorations (43). However, the inherent anatomical variability of natural human teeth resulted in larger standard deviations compared to *in vitro* models using typodont teeth or cast dies. Even CAD/CAM-milled extracted teeth cannot eliminate biological variability due to differences in donor age, oral history, and ultrastructural changes over time (44). The novel ST parameter offers an additional advantage by accounting for the actual size of the prepared surfaces, a factor previously difficult to quantify. This refinement provides a more individualized and clinically relevant assessment of adhesive interface adaptation compared to conventional luting space analysis alone.

No significant volumetric or surface changes were observed following artificial aging. Plastic deformation and volume changes in the adhesive luting composite (ALC) were not significant for both LS2 and RBC. Even though the image resolution of the applied micro-CT protocol was high (pixel size: 3.47–4.09 µm) no clinically relevant deformation of the restoration could be obtained; thus, hypothesis (a) was rejected. However, small changes with a limited extent could start at or below the available detection range, mainly driven by CPE and the visual inspection during artificial aging showed that parts of the luting composite were chipped off at the margins.

Interestingly, RBC restorations exhibited larger direct contact areas between the partial crown and prepared tooth surfaces (CPE, CPT, CPTR), which may indicate a larger amount of displacement of adhesive luting composite (ALC) due to slight deformation during seating, thus hypothesis (b) is supported. Mechanically, thin or absent adhesive layers may represent loss of connectivity between the two materials at these interfaces and could increase local stress concentrations at the material borders, raising the risk of marginal micro-leakage or failure initiation at the points of stress concentration, particularly in approximal inaccessible areas (45). However, such effects were not associated with mechanical failure in the present study. When recommended minimal restoration material thickness was maintained, no differences in survival rates were observed between LS2 and RBC. Should the restoration thickness fall below these recommendations, the prognosis may favor RBC due to its superior mechanical behavior at reduced thicknesses (21). Based on these findings, we propose the following hypothesis: that it is not the different configuration of the adhesive luting composite that is the critical factor, but rather the three-dimensionality of the material configuration of the partial crown (restoration configuration in relation to failure) or that the restoration configuration is a more critical factor for clinical load failure than the homogeneity of the adhesive luting composite.

There are various ways to report luting space thickness, resulting in diverse findings, regarding the optimal thickness with *in vitro* reports ranging from 23 to 406.5 µm (24). In general, it is distinguished between the thickness of the adhesive luting composite and the thickness of the luting space (fit, often measured without luting). Most clinical recommendations focus on the marginal gap rather than the luting space, as it is difficult to obtain in a clinical setting. Luting space thickness below 200 µm is preferred for better mechanical resistance and reduced polymerization shrinkage stress in *in vitro* studies (46). In this study the mean luting space thickness includes ALC (99.7% of luting space) with voids (0.3% of luting space), and the mean luting space thickness was approximately 200 µm for both LS2 and RBC, indicating the fit of both materials is almost identical. Volume changes from initial water uptake were ruled out after 90 days of water storage before scanning (9, 34, 47). During TCML, gap formation at the interfaces was expected as a result of further volume changes due to water absorption caused by the pump effect (48). Such effects may have been masked by excess luting composite chipping off at the edges, which in turn affected the overall volume of ALC (visible in Supplementary S2d). Thicker luting space areas were localized in central occlusal regions and drill compensation zones, where geometric constraints limit spacer control (49). Excessive luting space thickness beyond 500 µm may compromise bond strength due to tensile stress accumulation and reduce fracture resistance (50). Tensile strength decreases gradually with increasing luting space thickness, leading to a combination of cohesive and adhesive failure (51). With future advances in clinical imaging technology, the micro-CT approach and its translational potential may enable individualized quality control and monitoring of adhesive restorations directly in clinical practice. It could contribute to improved treatment outcomes and extended restoration longevity and it confirms data from gap detection with other imaging approaches [e.g., optical coherence tomography (27)].

Abrasion was visually detectable; however, the absolute material loss remained minimal relative to the total PC volume. Although VA did not reach statistical significance between LS2 and RBC after TCML the observed large effect size suggest a genuine, but small difference in wear behavior that the present sample size may have been insufficient to confirm statistically. This tendency may reflect differences in the material properties, such as hardness and matrix composition, of the two materials. Therefore, it warrants attention despite the lack of formal significance. These findings are consistent with those of (44), who reported similar abrasion volumes but found higher wear in RBC than LS2 using uniform premolar crowns with standardized cusp inclination and loading same points. It was suggested that wear is dependent on the cusp inclination of the restoration (52). Even though a reduced anatomical design was used, yet the inclination angles are not uniform for the specimens.

Several limitations must be acknowledged. Despite the high resolution of the micro-CT system, this represents an inherent detection threshold: interfacial gaps or voids below this scale remain undetectable. Furthermore, the radiopacity contrast between certain materials, particularly between adhesive and gaps, is inherently limited, as resin-based adhesives exhibit low x-ray attenuation and may appear as radiolucent voids indistinguishable from true gaps during segmentation. Filtering improved contrast but caused loss of very small structures (approximately 10 voxels in volume). Phase-contrast-enhanced micro-CT (PCE-micro-CT) at synchrotron facilities could offer significantly improved material differentiation; however, it is currently impractical due to limited availability. Furthermore, the present segmented dataset (2000*16 annotated slices per time point) could be used to train robust deep learning networks, that in the future would allow fully automated segmentation of this kind of datasets.

## Data Availability

The original contributions presented in the study are included in the article/supplementary material, further inquiries can be directed to the corresponding author/s.
