# Supplementary material for: 3D Evaluation of material-dependent volumetric and interfacial changes in CAD/CAM partial crowns: a semi-automated micro-CT approach
Source: Front Dent Med. 2026 Aug 18;7:1843676. doi: 10.3389/fdmed.2026.1843676 (PMC13529759; doi:10.3389/fdmed.2026.1843676)
Supplement: Supplementary file 1 [file Datasheet1.docx]

**Supplementary Material**

**Supplement 1:** Digital workflow (a) for semi-automated segmentation of large data using Biomedisa interpolation, and the following system specifications (b).

*1c) Stepwise detailed description of the workflow*: Segmentation was done by one trained operator on the same workstation and system.

Pre-processing: First a region of interest (ROI) was cut out of the original data set in Avizo 3D version 2021.1 (Thermo Fisher Scientific, Waltham MA, United States) cutting 20 x/y-planes above and below the edges of visible restorative material. Every twentieth equidistant x/y-plane micro-CT image was then pre-segmented manually without paying attention to voids (e.g. pores or gaps), resulting in 80 - 110 labeled slices and assigning six different materials (PC: LS2 or RBC, ALC, enamel, dentine, pulp and exterior) using the brush tool. In 8-bit data grey values range from 0 – 255. For LS2 the “Brush Masking” was set: PC 42-148, ALC 122-255, enamel 58-148, dentine 21-74, pulp 0-38; for RBC: PC 86-221, ALC 142-255, enamel 77-155, dentine 38-100, pulp 0 – 42; material islands up to 100 pixels were removed to clean the labels.

Interpolation: The open-source online platform Biomedisa (Lösel et al. 2020) was used for interpolation at default settings.

Post-processing (all materials except void): Afterwards a visual inspection was conducted for quality control. Post-processing was performed in Avizo. Cleaning included closing the materials by growing the voxels by five then shrinking them by five in the following order: 1) ALC 2) dentine 3) enamel 4) pulp 5) PC 6) exterior. Additionally, “Fill Holes” operator was used to close holes in all slices. Furthermore, islands up to the slice area of 100 pixels were automatically removed. Larger islands of isolated superfluous ALC on the PC exterior surface were removed manually in the 3D segmentation editor using the “Lasso” tool. A “Smoothing” module with a threshold of 3 was set for the 3D volume data.

Post-processing (void): Pores were segmented automatically using a threshold approach within luting space limits. Different filters were used on the image data to enhance contrast and edges of the voids. For LS2 the following filters were used:

1)	“Bilateral” filter with an x/y kernel size of 3, a similarity of 20 and bilateral filter mode.

2)	The “Non-Local Means” filter GPU standard with a search window of 10, local neighbourhood of 3, and similarity value of 0.6, adaptive true and automatic partition size enabled was used.

3)	The “Gaussian” filter kernel type separable, standard deviation of 1, and kernel size factor of 2 was used.

4)	A second “Bilateral” filter with the same settings as the Gaussian filter was used.

5)	An “Unsharp Masking” filter with and edge size 5, edge contrast 0.5 and brightness threshold of 0.

For RBC following filters were used:

1)	Three “Bilateral” filters each with a kernel size x and y of 3 and growing similarity first 20, then 100 and then 200

2)	“Non-Local Means” filter GPU standard with a search window 10, local neighborhood 3, similarity value 0.6, adaptive true and automatic partition size enabled

3)	“Median” filter with 10 iterations in iterative type

4)	An “Un-sharp masking” filter with edge size of 10, edge contrast of 0.5 and brightness threshold of 0 was applied.

Following filtering, the voids were segmented using a threshold within the luting space ROI: for LS2 gray values the optimal threshold was determined visually and set to 0-70, for RBC to 0-80. For cleaning each selection was shrinked by 4 voxels and afterwards grown by 4 voxels and added to a newly set material called void.

Analysis: Surface reconstruction was performed using the “Generate Surface” module (compactify enabled, minimum edge length set to 1, repeatable algorithm, and smoothing extent set to 5). After computing volume, surfaces and surface patches timestep 1 data (t1) was co-registered to timestep 2 data (t2) which served as reference data. The “VolRen” and the “Transform Editor” modules were used to pre-align t1 data manually and the “Register Image” module automatically completed registration. Images were then resampled with the “Resample Transformed Image” module (incl. a Lanczos interpolation) and data of t2 was used as reference. The new orientation was transferred to the labels using “Resample Transformed Image” module. Afterwards, PC data t2 was subtracted from t1 data with “Arithmetics” module, cleaning the labels by shrinking voxels by 1 and growing them again by 1, cutting away islands with the 3D lasso tool to show material loss of the PC during TCML. To enable the system to process large files they had to be downsampled to half of their original size for calculations on the luting space or a quarter for calculations on the crown material or matched data.

**Supplement 2:** Selection of non-filtered µCT images indicating voids and gaps (a and b) material demolition of the luting composite at the partial crown- (c), at the tooth (d), surplus of the partial crown (e). Size of the bar equals 1 mm.

**Supplement 3:** Alphabetic list of the R packages used in R (R Core Team, 2023, R: A Language and Environment for Statistical Computing. R Foundation for Statistical Computing, Vienna, Austria. https://www.R-project.org/); R code used as indicated in Supplement 4.

Wickham H, Averick M, Bryan J, Chang W, McGowan LD, François R, Grolemund G, Hayes A, Henry L, Hester J, Kuhn M, Pedersen TL, Miller E, Bache SM, Müller K, Ooms J, Robinson D, Seidel DP, Spinu V, Takahashi K, Vaughan D, Wilke C, Woo K, Yutani H (2019). "Welcome to the tidyverse." Journal of Open-Source Software, *4*(43), 1686. doi:10.21105/joss.01686

Fox J, Weisberg S (2019). An R Companion to Applied Regression, Third edition. Sage, Thousand Oaks CA. https://www.john-fox.ca/Companion.

Torchiano M (2020). effsize: Efficient Effect Size Computation. doi:10.5281/zenodo.1480624, R package version 0.8.1, https://CRAN.R-project.org/package=effsize

Champely S (2020). pwr: Basic Functions for Power Analysis. doi:10.32614/CRAN.package.pwr

Lenth R, Piaskowski J (2025). emmeans: Estimated Marginal Means, aka Least-Squares Means. doi:10.32614/CRAN.package.emmeans

**Supplement 4:** Detailed description of the R code of the statistical analysis (data preparation, processing, descriptive and inferential statistics, post-hoc power analysis) R code by Tobias Meißner.

1. # =============================================================================
2. # Statistical Analysis
3. # =============================================================================
4. # Study: Micro-CT Analysis of Partial Crown Restorations
5. # Analysis: Comparison of two material (LS2 and RBC) across two times (t1 and t2)
6. # Design: Repeated measures (paired samples for TIME, independent samples for GROUP)
7. # =============================================================================
9. # -----------------------------------------------------------------------------
10. # 1. ENVIRONMENT SETUP
11. # -----------------------------------------------------------------------------
13. rm(list **=** ls())
14. Sys.setenv(LANG **=** "en")
16. # Required packages
17. required_packages <**-** c(
18. "tidyverse",
19. "readxl",
20. "car",
21. "effsize",
22. "pwr"
23. )
25. **for** (pkg **in** required_packages) {
26. **if** (!require(pkg, character.only **=** TRUE)) install.packages(pkg)
27. library(pkg, character.only **=** TRUE)
28. }
30. # Note: This script was developed with R 4.5.2
32. # =============================================================================
33. # PART A: MICRO-CT MEASUREMENT ANALYSIS
34. # =============================================================================
36. # -----------------------------------------------------------------------------
37. # 2. DATA LOADING AND PREPARATION
38. # -----------------------------------------------------------------------------
40. # Load data from Excel file
41. data <**-** readxl::read_xlsx("path/to/data/Maintable.xlsx", sheet **=** "data")
42. used_columns <**-** readxl::read_xlsx("path/to/data/Maintable.xlsx", sheet **=** "USED_COLUMNS")
44. # Subset to relevant columns and convert to factors
45. data <**-** data **%**>**%**
46. dplyr::select(all_of(used_columns$COL_NAME)) **%**>**%**
47. dplyr::mutate(
48. GROUP **=** as.factor(GROUP),
49. TIME **=** as.factor(TIME),
50. MATERIAL **=** as.factor(MATERIAL)
51. )
53. # Rename measurement columns to paper naming convention
54. name_mapping <**-** c(
55. "VPC" **=** "vC",
56. "VV" **=** "vV",
57. "VLC/VLS" **=** "vTCG",
58. "CPE" **=** "pC_E",
59. "CPD" **=** "pC_D",
60. "SE" **=** "pSE",
61. "SD" **=** "pSD",
62. "SPC" **=** "csif1",
63. "ST" **=** "csif2",
64. "CPT" **=** "csif3",
65. "VA" **=** "awv",
66. "TLC/TLS" **=** "tTCG_mean",
67. "(PP)" **=** "rvV_vLC",
68. "CPTR" **=** "rcsif3_csif2",
69. "(RPT)" **=** "rcsif2_csif1",
70. "(VBL)" **=** "vTCG_BL"
71. )
73. data <**-** data **%**>**%** rename(any_of(name_mapping))
75. # Define measurement columns present in data
76. measurement_cols <**-** c("VPC", "VV", "VLC/VLS", "CPE", "CPD", "SE", "SD",
77. "SPC", "ST", "CPT", "VA", "TLC/TLS", "(PP)",
78. "CPTR", "(RPT)", "(VBL)")
79. measurement_cols <**-** measurement_cols[measurement_cols **%in%** names(data)]
81. # =============================================================================
82. # PART B: LUTING SPACE THICKNESS (TLS) DATA PROCESSING
83. # =============================================================================
84. # This section documents how mean luting space thickness (TLS) was calculated
85. # from histogram data. The resulting TLS values are then merged into the main
86. # dataset and analyzed using the same t-test paradigm as other measurements.
87. # -----------------------------------------------------------------------------
89. # Load histogram CSV files (thickness distribution per specimen)
90. file_list <**-** list.files("path/to/data/histograms/",
91. pattern **=** "*541_Histogram-Plot.csv",
92. full.names **=** TRUE,
93. recursive **=** TRUE)
95. df_main_combined <**-** lapply(file_list, function(x) {
96. df_temp <**-** read.csv(x, header **=** FALSE, sep **=** ',')
98. # Find delimiter row (where thickness resets to 0 after high values)
99. indices <**-** which(df_temp$V1 **==** 0 & c(FALSE, df_temp$V1[**-**length(df_temp$V1)] > 100))
100. df_temp <**-** df_temp[1:(indices[1]**-**1), ]
102. # Extract metadata from filename
103. df_temp <**-** df_temp **%**>**%**
104. dplyr::mutate(
105. DATASET **=** stringr::str_extract(basename(x), "^[^_]*_[^_]*_[^_]*"),
106. SAMPLE_ID **=** stringr::str_extract(basename(x), "^[^_]*"),
107. TIME **=** stringr::str_extract(basename(x), "(?<=_)[^_]*(?=_[^_]*)"),
108. INTERFACE **=** unlist(strsplit(basename(x), "_"))[3],
109. GROUP **=** stringr::str_sub(basename(x), 1, 1)
110. ) **%**>**%**
111. dplyr::rename(Thickness **=** V1, Count **=** V2) **%**>**%**
112. dplyr::select(DATASET, SAMPLE_ID, TIME, INTERFACE, GROUP, Thickness, Count)
114. **return**(df_temp)
115. })
117. df_main_combined <**-** do.call(rbind, df_main_combined) **%**>**%**
118. # Exclude measurements below 50µm threshold
119. dplyr::mutate(Count **=** dplyr::if_else(Thickness < 50, 0, Count))
121. # Aggregate to specimen-level: calculate weighted mean thickness
122. specimen_summary <**-** df_main_combined **%**>**%**
123. group_by(SAMPLE_ID, GROUP, TIME) **%**>**%**
124. summarise(
125. total_voxels **=** sum(Count),
126. mean_thickness **=** weighted.mean(Thickness, Count),
127. .groups **=** "drop"
128. )
130. # Merge TLS (mean_thickness) into main dataset
131. data <**-** data **%**>**%**
132. mutate(specimen_key **=** paste0(stringr::str_sub(GROUP, 1, 1),
133. gsub(".*_(\\d+)_.*", "\\1", SAMPLE_ID))) **%**>**%**
134. left_join(specimen_summary **%**>**%** select(SAMPLE_ID, TIME, mean_thickness),
135. by **=** c("specimen_key" **=** "SAMPLE_ID", "TIME" **=** "TIME")) **%**>**%**
136. select(**-**specimen_key) **%**>**%**
137. rename(TLS **=** mean_thickness)
139. # Add TLS to measurement columns
140. measurement_cols <**-** c(measurement_cols, "TLS")
142. # -----------------------------------------------------------------------------
143. # 3. DESCRIPTIVE STATISTICS
144. # -----------------------------------------------------------------------------
146. descriptive_stats <**-** data **%**>**%**
147. group_by(GROUP, TIME) **%**>**%**
148. summarise(across(all_of(measurement_cols),
149. list(n **=** ~sum(!**is**.na(.)),
150. mean **=** ~mean(., na.rm **=** TRUE),
151. sd **=** ~sd(., na.rm **=** TRUE),
152. min **=** ~min(., na.rm **=** TRUE),
153. max **=** ~max(., na.rm **=** TRUE),
154. median **=** ~median(., na.rm **=** TRUE)),
155. .names **=** "{.col}_{.fn}"),
156. .groups **=** "drop") **%**>**%**
157. pivot_longer(cols **=** **-**c(GROUP, TIME),
158. names_to **=** c("Measurement", "Statistic"),
159. names_pattern **=** "(.*)_(.*)",
160. values_to **=** "Value") **%**>**%**
161. mutate(across(where(**is**.numeric), ~round(., 4))) **%**>**%**
162. pivot_wider(names_from **=** Statistic, values_from **=** Value) **%**>**%**
163. arrange(Measurement)
165. # -----------------------------------------------------------------------------
166. # 4. ASSUMPTION TESTING
167. # -----------------------------------------------------------------------------
169. # 4.1 Normality Test (Shapiro-Wilk)
171. normality_results <**-** data **%**>**%**
172. pivot_longer(cols **=** all_of(measurement_cols),
173. names_to **=** "Measurement",
174. values_to **=** "Value") **%**>**%**
175. group_by(Measurement, GROUP, TIME) **%**>**%**
176. summarise(
177. n **=** sum(!**is**.na(Value)),
178. W **=** shapiro.test(Value)$statistic,
179. p_value **=** round(shapiro.test(Value)$p.value, 4),
180. .groups **=** "drop"
181. ) **%**>**%**
182. mutate(Normal **=** ifelse(p_value > 0.05, "Yes", "No")) **%**>**%**
183. arrange(Measurement)
185. # 4.2 Homogeneity of Variance (Levene's Test)
187. levene_results <**-** data **%**>**%**
188. pivot_longer(cols **=** all_of(measurement_cols),
189. names_to **=** "Measurement",
190. values_to **=** "Value") **%**>**%**
191. group_by(TIME, Measurement) **%**>**%**
192. summarise(
193. F_value **=** round(car::leveneTest(Value, GROUP)$"F value"[1], 4),
194. p_value **=** round(car::leveneTest(Value, GROUP)$"Pr(>F)"[1], 4),
195. .groups **=** "drop"
196. ) **%**>**%**
197. mutate(Equal_Variance **=** ifelse(p_value > 0.05, "Yes", "No")) **%**>**%**
198. pivot_wider(names_from **=** TIME, values_from **=** c(F_value, p_value, Equal_Variance)) **%**>**%**
199. arrange(Measurement)
201. # -----------------------------------------------------------------------------
202. # 5. INFERENTIAL STATISTICS
203. # -----------------------------------------------------------------------------
205. # 5.1 Paired t-Test (TIME comparison within each GROUP)
207. time_levels <**-** levels(data$TIME)
208. time_col1 <**-** time_levels[1]
209. time_col2 <**-** time_levels[2]
211. # Reshape data for paired analysis
212. paired_data <**-** data **%**>**%**
213. mutate(SUBJECT_ID **=** gsub("_t[0-9]+", "", SAMPLE_ID)) **%**>**%**
214. pivot_longer(cols **=** all_of(measurement_cols),
215. names_to **=** "Measurement",
216. values_to **=** "Value") **%**>**%**
217. pivot_wider(names_from **=** TIME,
218. values_from **=** Value,
219. id_cols **=** c(SUBJECT_ID, GROUP, TOOTH, MATERIAL, Measurement))
221. paired_ttest_results <**-** paired_data **%**>**%**
222. group_by(GROUP, Measurement) **%**>**%**
223. group_modify(~ {
224. x <**-** .x[[time_col1]]
225. y <**-** .x[[time_col2]]
226. tibble(
227. n **=** sum(!**is**.na(x) & !**is**.na(y)),
228. mean_diff **=** round(mean(x **-** y, na.rm **=** TRUE), 4),
229. t_statistic **=** tryCatch(round(t.test(x, y, paired **=** TRUE)$statistic, 4), error **=** function(e) NA_real_),
230. df **=** tryCatch(t.test(x, y, paired **=** TRUE)$parameter, error **=** function(e) NA_real_),
231. p_value **=** tryCatch(round(t.test(x, y, paired **=** TRUE)$p.value, 4), error **=** function(e) NA_real_),
232. Cohens_d **=** tryCatch(round(effsize::cohen.d(x, y, paired **=** TRUE, na.rm **=** TRUE)$estimate, 3), error **=** function(e) NA_real_)
233. )
234. }) **%**>**%**
235. ungroup() **%**>**%**
236. arrange(Measurement, GROUP)
238. # 5.2 Independent t-Test (GROUP comparison at each TIME)
240. independent_ttest_results <**-** data **%**>**%**
241. pivot_longer(cols **=** all_of(measurement_cols),
242. names_to **=** "Measurement",
243. values_to **=** "Value") **%**>**%**
244. group_by(TIME, Measurement) **%**>**%**
245. summarise(
246. n_C **=** sum(GROUP **==** "C" & !**is**.na(Value)),
247. n_D **=** sum(GROUP **==** "D" & !**is**.na(Value)),
248. mean_C **=** round(mean(Value[GROUP **==** "C"], na.rm **=** TRUE), 4),
249. mean_D **=** round(mean(Value[GROUP **==** "D"], na.rm **=** TRUE), 4),
250. t_statistic **=** tryCatch(round(t.test(Value ~ GROUP)$statistic, 4), error **=** function(e) NA_real_),
251. df **=** tryCatch(round(t.test(Value ~ GROUP)$parameter, 2), error **=** function(e) NA_real_),
252. p_value **=** tryCatch(round(t.test(Value ~ GROUP)$p.value, 4), error **=** function(e) NA_real_),
253. Cohens_d **=** tryCatch(round(effsize::cohen.d(Value ~ GROUP, na.rm **=** TRUE)$estimate, 3), error **=** function(e) NA_real_),
254. .groups **=** "drop"
255. ) **%**>**%**
256. arrange(Measurement, TIME)
258. # =============================================================================
259. # PART C: POST-HOC POWER ANALYSIS
260. # =============================================================================
261. # This section addresses sample size considerations by calculating:
262. # 1. Achieved power for each measurement given observed effect sizes
263. # 2. Minimum detectable effect (MDE) with 80% power
264. # -----------------------------------------------------------------------------
266. # Target measurements for power analysis (as reported in paper)
267. target_vars <**-** c("TLS", "CPTR", "CPD", "CPE", "VLC/VLS")
269. # -----------------------------------------------------------------------------
270. # 6. POWER ANALYSIS: INDEPENDENT T-TESTS (GROUP COMPARISONS)
271. # -----------------------------------------------------------------------------
273. power_independent <**-** data **%**>**%**
274. pivot_longer(cols **=** all_of(target_vars),
275. names_to **=** "Measurement",
276. values_to **=** "Value") **%**>**%**
277. group_by(TIME, Measurement) **%**>**%**
278. summarise(
279. n_per_group **=** n() **/** 2,
280. Cohens_d **=** abs(effsize::cohen.d(Value ~ GROUP, na.rm **=** TRUE)$estimate),
281. .groups **=** "drop"
282. ) **%**>**%**
283. rowwise() **%**>**%**
284. mutate(achieved_power **=** pwr::pwr.t.test(n **=** n_per_group, d **=** Cohens_d,
285. sig.level **=** 0.05, type **=** "two.sample")$power) **%**>**%**
286. ungroup()
288. # -----------------------------------------------------------------------------
289. # 7. POWER ANALYSIS: PAIRED T-TESTS (TIME COMPARISONS)
290. # -----------------------------------------------------------------------------
292. paired_power_data <**-** data **%**>**%**
293. mutate(SUBJECT_ID **=** gsub("_t[0-9]+", "", SAMPLE_ID)) **%**>**%**
294. pivot_longer(cols **=** all_of(target_vars),
295. names_to **=** "Measurement",
296. values_to **=** "Value") **%**>**%**
297. pivot_wider(names_from **=** TIME,
298. values_from **=** Value,
299. id_cols **=** c(SUBJECT_ID, GROUP, Measurement))
301. power_paired <**-** paired_power_data **%**>**%**
302. group_by(GROUP, Measurement) **%**>**%**
303. summarise(
304. n_pairs **=** n(),
305. Cohens_d **=** abs(effsize::cohen.d(.data[[time_col1]], .data[[time_col2]],
306. paired **=** TRUE, na.rm **=** TRUE)$estimate),
307. .groups **=** "drop"
308. ) **%**>**%**
309. rowwise() **%**>**%**
310. mutate(achieved_power **=** pwr::pwr.t.test(n **=** n_pairs, d **=** Cohens_d,
311. sig.level **=** 0.05, type **=** "paired")$power) **%**>**%**
312. ungroup()
314. # -----------------------------------------------------------------------------
315. # 8. MINIMUM DETECTABLE EFFECT SIZE (80% power, α = 0.05, n = 8)
316. # -----------------------------------------------------------------------------
318. mde_independent <**-** pwr::pwr.t.test(n **=** 8, sig.level **=** 0.05, power **=** 0.80, type **=** "two.sample")$d
319. mde_paired <**-** pwr::pwr.t.test(n **=** 8, sig.level **=** 0.05, power **=** 0.80, type **=** "paired")$d
